# Cryo-EM structure of the human THIK-1 K2P K^+^ channel reveals a lower Y gate regulated by lipids and anesthetics

**DOI:** 10.1038/s41594-025-01497-6

**Published:** 2025-02-26

**Authors:** Karin E. J. Rödström, Bisher Eymsh, Peter Proks, Mehtab S. Hayre, Sönke Cordeiro, Edward Mendez-Otalvaro, Christian Madry, Anna Rowland, Wojciech Kopec, Simon Newstead, Thomas Baukrowitz, Marcus Schewe, Stephen J. Tucker

**Affiliations:** 1 Kavli Institute for Nanoscience Discovery, University of Oxford, Oxford, UK; 2 Clarendon Laboratory, Department of Physics, University of Oxford, Oxford, UK; 3 Department of Biochemistry, University of Oxford, Oxford, UK; 4 Institute of Physiology, Kiel University, Kiel, Germany; 5 Computational Biomolecular Dynamics Group, Max Planck Institute, Göttingen, Germany; 6 Institute of Neurophysiology, Charité-Universitätsmedizin Berlin, Berlin, Germany; 7 Cerevance Ltd, Cambridge Science Park, Cambridge, UK; 8 Department of Chemistry, Queen Mary University of London, London, UK; 9 OXION Initiative in Ion Channels and Disease, University of Oxford, Oxford, UK

**Keywords:** Ion transport, Cryoelectron microscopy

## Abstract

THIK-1 (*KCNK13*) is a halothane-inhibited and anionic-lipid-activated two-pore domain (K2P) K^+^ channel implicated in microglial activation and neuroinflammation, and a current target for the treatment of neurodegenerative disorders, for example Alzheimer’s disease and amyothropic lateral sclerosis (ALS). However, compared to other K2P channels, little is known about the structural and functional properties of THIK-1. Here we present a 3.16-Å-resolution cryo-EM structure of human THIK-1 that reveals several distinct features, in particular, a tyrosine in M4 that contributes to a lower ‘Y gate’ that opens upon activation by physiologically relevant G-protein-coupled receptor and lipid signaling pathways. We demonstrate that linoleic acid bound within a modulatory pocket adjacent to the filter influences channel activity, and that halothane inhibition involves a binding site within the inner cavity, both resulting in conformational changes to the Y gate. Finally, the extracellular cap domain contains positively charged residues that line the ion exit pathway and contribute to the distinct biophysical properties of this channel. Overall, our results provide structural insights into THIK-1 function and identify distinct regulatory sites that expand its potential as a drug target for the modulation of microglial function.

## Abstract (web summary)

In this study, Rödström et al. use cryo-EM to reveal a range of structural features in THIK-1 K^+^ channel and demonstrate how these features define many functional properties of the channel, thus expanding its potential as a drug target.

## Main

K2P channels are a distinct subset of K^+^ channels that assemble as dimers to form a pseudo-tetrameric central pore. The 15 human K2P (*KCNK*) channels respond to various physical, chemical and biological signals, regulating resting membrane potential and coupling these inputs to changes in cellular electrical activity^1,2^. Their functional roles, especially in the central and peripheral nervous system, make them key pharmacological targets, and their dysfunction is linked to several diseases and neurodevelopmental disorders^3^.

THIK-1, encoded by *KCNK13*, is broadly distributed in the central nervous system in rodents^4^, but its expression in humans is primarily restricted to microglia^5^, the brain’s innate immune cells. Microglia protect the brain from injury and invading pathogens, becoming ‘activated’ to trigger an immunological response to contain neuronal damage^6–8^. However, in some cases, microglia can adopt disease-exacerbating states of activation, leading to neuroinflammation, a key driver of neurodegenerative disorders such as Alzheimer’s disease, Parkinson’s disease and ALS. THIK-1 channels are crucial for the release of proinflammatory cytokines during the activation of human microglia, and *KCNK13* is upregulated in both animal models of neurodegeneration and Alzheimer’s disease itself^9^. THIK-1 inhibitors slow neurodegeneration in animal models^10^, and THIK-1 activators could promote immune surveillance of the brain parenchyma when microglial immune surveillance is impaired. Consequently, this channel is a promising therapeutic target for the modulation of microglial function^11^, and clinical trials of a THIK-1 inhibitor are underway^10,12^.

Compared with many K2P channels, the structural and functional properties of THIK channels are poorly understood. Identified in 2000 (ref. ^4^) as the TWIK-related-halothane-inhibited K^+^ channels, THIK-1 and THIK-2, this subfamily has received limited study despite robust functional expression of THIK-1 in heterologous systems, leaving the structural basis for its biophysical and functional properties largely unclear. The other member of this subfamily, THIK-2, shares 62% sequence identity with THIK-1 and can coassemble with THIK-1 to form heteromeric channels^13^. However, THIK-2 is largely retained in the endoplasmic reticulum (ER), limiting its functional analysis; THIK-2 currents have been measured only by removing its ER-retention motif to promote trafficking to the cell surface and/or the introduction of substitutions in the transmembrane helices that have an activatory gain-of-function effect^14,15^.

Recent studies have demonstrated that THIK-1 can be transiently activated by both G_i/o_- and G_q_-coupled receptor pathways^16^ and directly activated by anionic lipids such as phosphatidylinositol 4,5-bisphosphate (PIP_2_) and oleoyl-CoA^17^. In addition, THIK-1 exhibits direct inhibition by the phosphodiesterase inhibitor, 3-isobutyl-1-methyl-xanthine (IBMX)^18^, as well as several small-molecule inhibitors in development as therapeutics^10,12^. However, the detailed mechanisms of this pharmacology are not well understood, nor are its biophysical properties. This is because the single-channel conductance of THIK-1 is very low (<5 pS) with only brief flickery openings (<0.5 ms), thereby limiting detailed biophysical analysis of its gating^19^. Given the importance of THIK-1 as a potential therapeutic target, a better understanding of its structural and functional properties is warranted.

Here we present a cryogenic electron microscopy (cryo-EM) structure of human THIK-1 at 3.16-Å resolution in combination with a detailed functional analysis. This study reveals several unique structural features that provide important insights into the molecular basis of THIK-1 function, its regulation by lipids and volatile anesthetics, and its suitability as a drug target for the regulation of microglial function.

## Results and discussion

### THIK-1 cryo-EM structure reveals unique structural features

**Fig. 1:**
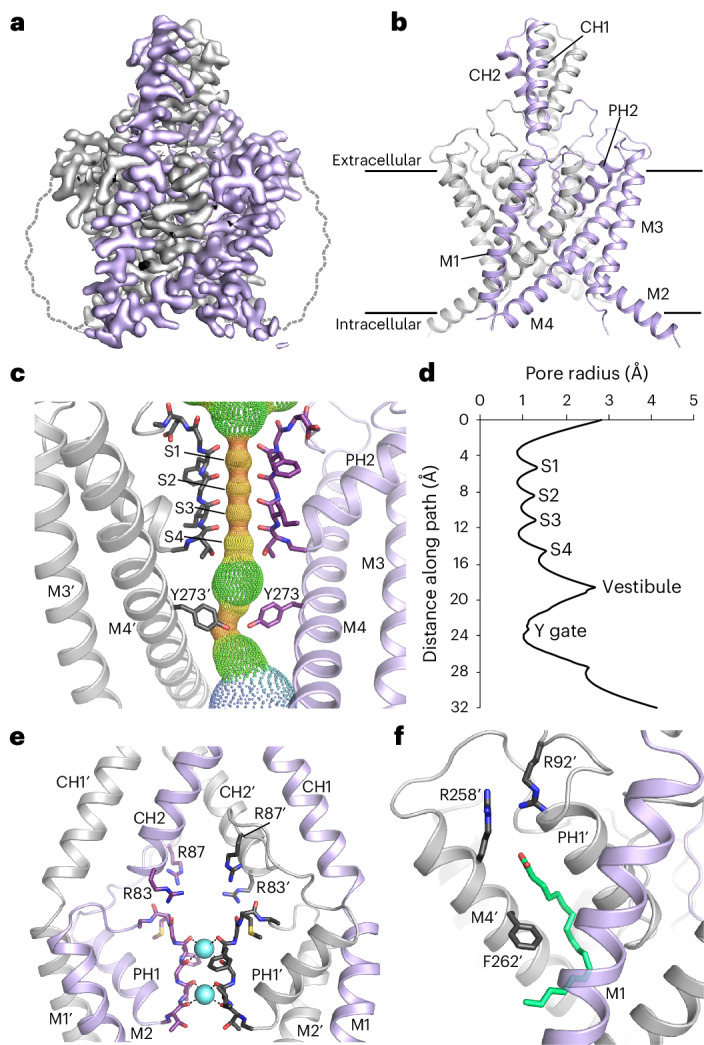
Structure of THIK-1. **a**, Sharpened cryo-EM map viewed from the side with the density for THIK-1 channel subunits in gray and purple. The approximate position of the detergent micelle is outlined as a dashed gray line. **b**, Structure of THIK-1 colored as in **a**, with the M1–M4 transmembrane helices, and the pore and cap helices labeled. **c**, HOLE profile through the channel pore with the selectivity filter (S1–S4 sites) and the constriction formed by the lower tyrosine 273 (Y gate) site highlighted. For clarity, M1, M2 and PH1 have been hidden. **d**, Pore radius of the THIK-1 channel interior as a distance function along the ion-permeation pathway. **e**, Ion-exit pathway at the extracellular site of the selectivity filter is lined by a cluster of positively charged residues (R83 and R87 from both subunits). M3, M4 and PH2 have been hidden for clarity. **f**, The K2P modulator pocket, showing a lipid bound at the inter-subunit interface of M4, PH1 and M1. Key residues in close proximity are highlighted as sticks. Source data

**Extended Data Fig. 1:**
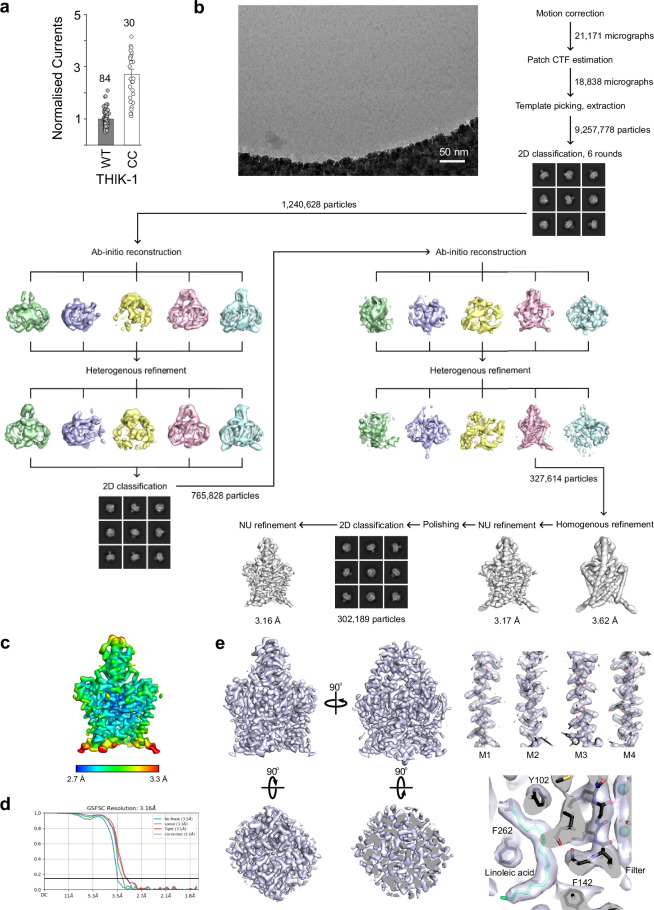
Cryo-EM processing pipeline for THIK-1. **a**, The cryoEM construct (CC) used for structural studies remains functionally active; whole-cell currents (recorded at +50 mV) are normalised to WT THIK-1. All values are given as mean ± s.e.m with number (n) of individual recordings indicated above the bars. **b**, CryoEM imaging processing workflow. **c**, Local resolution estimation of the unsharpened map, determined within cryoSPARC. **d**, Gold standard FSC curve for resolution estimation, calculated within cryoSPARC. **e**, The sharpened map, overall views from the sides, bottom and slice through the top. Maps 2.6 Å around the M1-M4 helices and a close up view of the map in the lipid binding site.

Similar to previous approaches used for K2P channels, we first removed the predicted unstructured carboxy-terminal region of THIK-1 and confirmed that this truncated channel (Gly9–Gly297) was functionally active. The channel was expressed in insect cells and purified for single-particle cryo-EM. The resulting structure was resolved to 3.16-Å resolution (Fig. 1, Table 1 and Extended Data Fig. 1).

**Table 1:** Cryo-EM data collection, refinement and validation statistics

	THIK-1 (EMDB-50741), (PDB 9FT7)
**Data collection and processing**	
Magnification	×105,000
Voltage (kV)	300
Electron exposure (e^–^/Å^2^)	42.54
Defocus range (μm)	−1 to −2.3
Pixel size (Å)	0.832
Symmetry imposed	*C*_2_
Initial particle images (no.)	9,257,778
Final particle images (no.)	302,189
Map resolution (Å)	3.53
FSC threshold	0.5
Map resolution (Å)	3.16
FSC threshold	0.143
Map resolution range (Å)	2.72–8.56
**Refinement**	
Initial model used (PDB code)	De novo
Model resolution (Å)	3.29
FSC threshold	0.5
Model resolution range (Å)	2.72–8.56
Map sharpening *B* factor (Å^2^)	−140
Model composition	
Non-hydrogen atoms	4,016
Protein residues	508
Potassium ions	2
Linoleic acid	2
*B* factors (Å^2^)	
Protein	64.73
Potassium ion	23.63
Linoleic acid	48.43
R.m.s. deviations	
Bond lengths (Å)	0.007
Bond angles (°)	1.064
**Validation**	
MolProbity score	0.85
Clashscore	1.26
Poor rotamers (%)	0
Ramachandran plot	
Favored (%)	98.82
Allowed (%)	1.18
Disallowed (%)	0

The overall fold of the channel is similar to that of all other known K2P channel structures; it forms a domain-swapped homodimer, with each subunit containing four transmembrane helices (M1–M4), two pore helices (PH1 and PH2), two selectivity filter (SF) motifs (SF1 and SF2) and two extracellular cap-forming helices (CH1 and CH2). However, unlike many K2Ps, it lacks a cysteine at the relevant position at the apex, preventing the formation of an interchain disulphide bond. The SF adopts a near four-fold symmetry and is similar to that of most other K2P channels, but the intracellular M2–M3 loop (Arg166–Gly189) is longer than that in most K2P channels and was not resolved, presumably owing to its conformational heterogeneity (Fig. 1a,b).

Despite these similarities, several unpredicted structural features were identified that help explain the unique functional properties of this channel. These include a lower constriction of the permeation pathway in the inner cavity created by the M2 and M4 pore-lining helices (Fig. 1c,d), a cap domain with positively charged residues lining the extracellular ion exit pathways (Fig. 1e) and a single-chain lipid bound in the cryptic K2P modulator pocket adjacent to the SF (Fig. 1f).

### A lower ‘Y gate’

**Fig. 2:**
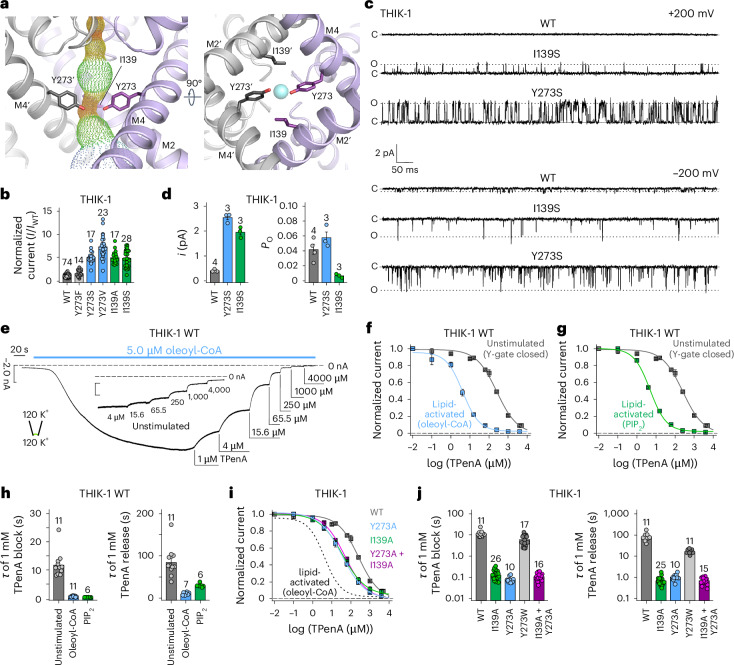
THIK-1 Y gate regulated by lipids. **a**, The Y gate viewed from the side (M2′ hidden for clarity) and from the bottom, showing that residue I139 is on the same horizontal level as Y273 and is part of the constriction formed by the Y gate. **b**, Relative whole-cell current amplitudes of WT THIK-1 and channels with substitutions in the Y gate. All currents (*I*) are normalized to that of the WT channel (*I*_WT_). **c**, Cell-attached recordings of 1-s duration at ±200 mV, containing single WT, I139S and Y273S THIK-1 channels, as indicated. The closed (c) and open channel (o) levels are shown. **d**, Comparison of single-channel open probability (*P*_o_) and single channel current amplitude (*i*) of WT, I139S and Y273S THIK-1 channels in cell-attached patches at −200mV. **e**, Representative macroscopic recording at −80 mV from an inside-out patch containing WT THIK-1 channels with symmetrical K^+^ concentrations (120 mM) at pH 7.4. Channel currents were activated by 5 µM oleoyl-CoA applied to the intracellular side of the membrane and then inhibited in a dose-dependent manner by TPenA. Inlay shows equal inhibition with TPenA at the unstimulated, basal state of the channels. **f**,**g**, Analysis of the affinity for TPenA from recordings in **e**, showing increased TPenA sensitivity after either oleoyl-CoA activation (**f**) or PIP_2_ activation (**g**). **h**, Analysis of TPenA kinetics for the block and release of WT THIK-1 in unstimulated and lipid-activated states. **i**,**j**, Analysis of apparent TPenA affinity (**i**) and kinetics (**j**) for WT and THIK-1 mutants from recordings as in the inlay in **e**. Throughout the graphs, all values are given as mean ± s.e.m., with the number of experiments or recordings (*n*) shown above the bars. Source data

Several K2P channels gate exclusively within the SF and do not possess a lower cytoplasmic gate. However, the structure of THIK-1 reveals a major constriction within the inner cavity that is created by the interaction of bulky side chains on both the M2 and M4 helices. Specifically, a tyrosine (Y273) on each M4 helix points inwards and, along with I139 on M2, occludes the pore (Figs. 1c and 2a). This constriction differs from the lower ‘X gate’ found in TASK channels which involves only residues on M4. Also, the Y gate is positioned slightly higher than the X gate, thus creating an even smaller inner vestibule below the SF in THIK-1. The Y gate completely occludes the permeation pathway in THIK-1 (Fig. 1d), so this conformation is predicted to be closed and non-conductive.

To examine the functional relevance of this constriction, we measured the activity of the Y273S and I139S mutations in this putative gating motif of THIK-1. We expressed these substitutions in *Xenopus* oocytes and found that both mutants produced markedly increased whole-cell K^+^ currents compared with WT THIK-1, indicating that these residues are important in controlling channel activity (Fig. 2b).

We next examined their effects in more detail at the single-channel level. Consistent with previous studies^18,19^, detecting single-channel activity for WT THIK-1 in cell-attached patches with symmetrical K^+^ concentrations (140 mM) proved challenging. Small flickery openings were reliably resolved only at very negative potentials (−200 mV), not at positive potentials (up to +200 mV) (Fig. 2c). This suggests that, at positive potentials, openings are either so fast that they are filtered out or have conductance too low to be resolved by the recording system.

**Extended Data Fig. 2:**
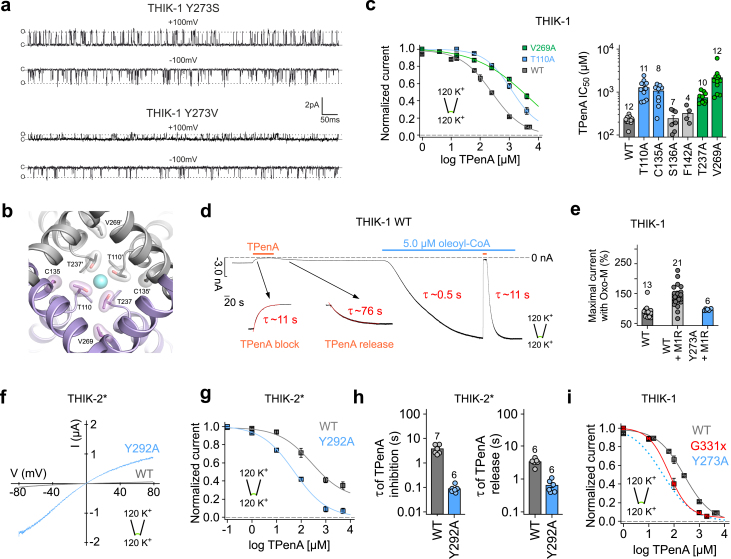
Y-gating and TPenA inhibition in THIK channels. **a**, Cell-attached recordings of 1 s duration at ± 100 mV containing single Y273S and Y273V THIK 1 channels, as indicated. The closed (c) and open channel (o) levels are indicated. **b**, Bottom view towards the selectivity filter with residues involved in TPenA binding highlighted. The K^+^ ion in the filter is represented in cyan. **c**, TPenA dose-response curves for WT (gray), T110A (blue) and V269 (green) mutant THIK-1 channels. TPenA IC_50_ values for WT and indicated mutants. **d**, Representative recording at −80 mV from an inside out patch of *Xenopus* oocyte expressing WT THIK-1 channels with symmetrical K^+^ concentrations (120 mM [K^+^]_ext_/120 mM [K^+^]_int._) at pH 7.4. Channel currents were inhibited with 1 mM TPenA in the absence and presence of 5 µM oleoyl-CoA. **e**, Reduced GqPCR-mediated activation of Y273A mutant channels compared to WT THIK-1 (coexpressed with hM1R and activated by 10 μM Oxo-M). **f**, Representative recording from inside out patches containing either THIK-2* (gray trace) or THIK-2* Y292A channels (blue trace) with symmetrical [K^+^] (120 mM [K^+^]_ext_/120 mM [K^+^]_int._) at pH 7.4. **g, h**, TPenA dose-response curves (f) and block/release kinetics (g) for THIK-2* (gray) and THIK-2* Y292A (blue) mutant channels, respectively. **i**, TPenA dose-response curves for WT (gray) and G331x mutant THIK-1 channels. Note, dashed line indicates TPenA dose-response curve for THIK-1 Y273A channels. All values are given as mean ± s.e.m with number (n) of individual recordings indicated above the bars. Source data

By contrast, both THIK-1-Y273S and THIK-1-I139S produced a marked increase in the single-channel conductance (~6.5-fold greater than WT for THIK-1-Y273S, and ~5-fold greater for THIK-1-I139S at −200 mV), as well as detectable openings at positive membrane potentials (Fig. 2c,d and Extended Data Fig. 2a). Notably, compared with WT THIK-1 at −200 mV, the Y273S mutant had no obvious effect on channel open probability (*P*_o_), whereas the *P*_o_ of THIK-1-I139S was reduced owing to an increase in the stability of its long closed states (Fig. 2c,d).

We next examined the effect of a more conservative substitution at this position: THIK-1-Y273F produced only a ~1.5-fold increase in whole-cell currents (Fig. 2b), with single-channel properties indistinguishable from those of WT THIK-1 at −200 mV (*i* = 0.47 ± 0.09 pA, *n* = 3; *P*_o_ = 0.058 ± 0.04, *n* = 3). By contrast, the Y273V mutant produced an increase in whole-cell current of more than sevenfold (Fig. 2b), which was also matched by a substantial rise in single-channel conductance (*i* = −2.75 ± 0.01 pA at −200 mV; *n* = 3) (Extended Data Fig. 2a).

Overall, this demonstrates that the effects of these substitutions are complex and affect both K^+^ permeation and the dynamics of gating. The Y273 side chain therefore plays an important role in both these processes, and we hereafter refer to this structural motif as the Y gate.

### The Y gate opens upon lipid and G-protein-coupled receptor activation

If this structural motif is part of a physiologically relevant gating mechanism, then it should open and close in response to regulatory inputs. It has previously been shown that THIK-1 is directly activated by polyanionic lipids, such as PIP_2_ and oleoyl-CoA^17^. We therefore examined whether lipid regulation directly influences the Y gate by measuring accessibility of pore blockers to the inner cavity above the Y gate.

Like most K_2P_ channels, THIK-1 can be inhibited by intracellular application of tetrapentylammonium (TPenA). Consistent with this, we found that intracellular application of TPenA to excised patches from oocytes expressing THIK-1 produced inhibition, with a half-maximal inhibitory concentration (IC_50_) of 225 ± 13 µM (*n* = 12), and mutating key residues immediately below the filter of THIK-1 in the predicted consensus binding site for TPenA (T110A; C135A; T237A; V269A) reduced this inhibition (IC_50_ = 1,284 ± 174 (*n* = 11), 1,063 ± 140 (*n* = 8), 751 ± 69 (*n* = 10) and 2,203 ± 472 µM (*n* = 10), respectively) (Extended Data Fig. 2b,c). This confirmed that, like other K2P channels, THIK-1 undergoes direct pore block by TPenA just below the SF. Strikingly, the kinetics of TPenA block (*τ*_block_) and release (*τ*_release_) were the slowest of all the K2P channels examined so far (*τ*_block_ = 12 ± 1 s and *τ*_release_ = 85 ± 11 s; *n* = 11) (Extended Data Fig. 2d), consistent with the fact that the Y gate clearly restricts access of TPenA to its binding site below the SF.

To address whether lipid activation regulates the Y gate, we next measured the properties of TPenA inhibition in lipid-activated THIK-1 WT channels. We found that TPenA sensitivity was markedly increased by both oleoyl-CoA activation (IC_50_ = 3.9 ± 0.3; *n* = 22) and 10 µM PIP_2_ activation (IC_50_ = 4.6 ± 0.2; *n* = 5) (Fig. 2e–g). Furthermore, both the kinetics and efficacy of TPenA block were increased by lipid activation (for oleoyl-CoA, *τ*_block_ = 0.5 ± 0.1 s, *τ*_release_ = 11 ± 1 s; for PIP_2_ *τ*_block_ = 0.31 ± 0.02 s, *τ*_release_ = 30 ± 2 s; *n* ≥ 6) (inhibition of the WT channel with 15.6 µM TPenA was 11 ± 1%, in oleoyl-CoA it was 78 ± 1% and in PIP_2_ it was 74 ± 1%; *n* ≥ 5) (Fig. 2h). In addition, the sensitivity of TPenA inhibition was increased by activatory mutations in the Y gate (THIK-1-Y273A: IC_50_ = 38 ± 7, *n* = 6; THIK-1-I139A: IC_50_ = 38 ± 3, *n* = 13), as were the kinetics of TPenA inhibition. As a control, substitution with a bulky aromatic (THIK-1-Y273W) had little to no effect on these kinetics (Fig. 2i,j). These results therefore demonstrate that activation by anionic lipids dynamically changes the access of TPenA to its binding site by opening the Y gate.

THIK-1 can also be activated by Gq-coupled receptor pathways^16^, so we next examined the effect of substitutions in the Y gate on activation by the human muscarinic receptor (hM1-R). Consistent with previous studies^16^, WT THIK-1 exhibited ~50% activation by 10 μM Oxo-M, whereas no activation was observed in the absence of hM1-R or with THIK-1-Y273A (Extended Data Fig. 2e). Together, these results indicate that a variety of physiologically relevant signaling mechanisms converge on the Y gate to regulate THIK-1 channel activity.

The residues that comprise the Y gate are also conserved in the related THIK-2 channel. Using an amino-terminal ER-retention mutant (THIK-2*) that traffics to the membrane^20^, we found that mutation of the Y gate (THIK-2*-Y292A) also produced a gain-of-function effect that affected both the sensitivity and kinetics of TPenA inhibition (Extended Data Fig. 2f–h).

Furthermore, it has recently been shown that THIK-1 channels are involved in apoptotic processes, such as cell shrinkage, through caspase-8 (CASP8), which cleaves the carboxy terminus of THIK-1 (ref. ^21^). Notably, truncation of the channel at this CASP8 cleavage site (G331x) also resulted in a gain-of-function effect and increased TPenA sensitivity (G331x IC_50_ = 79 ± 17; *n* = 7) compared with the WT channel (Extended Data Fig. 2i), suggesting that CASP8 cleavage increases channel activity by promoting Y-gate opening. Overall, these results clearly demonstrate that the Y-gate motif integrates a variety of physiologically relevant signals into a gating process that is conserved in this subfamily of K2P channels.

### Halothane inhibition also acts through the Y gate

**Fig. 3:**
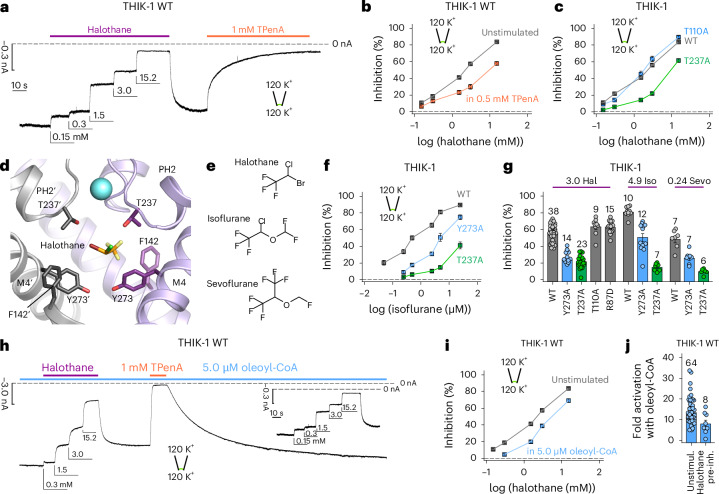
Inhibition by volatile anesthetics involves both the filter and Y gate. **a**, Representative recording at −80 mV from an inside-out patch containing WT THIK-1 channels with symmetrical K^+^ concentrations (120 mM) at pH 7.4. Channel currents were inhibited in a dose-dependent manner with increasing concentrations of halothane applied to the intracellular side of the membrane. Halothane effects can be washed and recovered and the currents inhibited with TPenA. **b**, Analysis of halothane inhibition for THIK-1 WT from recordings as in **a**, in the absence (gray) and presence (orange) of 0.5 mM TPenA, which produces an ~80 % block of initial currents. **c**, Analysis of halothane inhibition from recordings as in **a** for WT THIK-1 and indicated mutant channels. **d**, THIK-1 with halothane docked in the vestibule. Residues in close proximity are highlighted as sticks. For clarity, residues 121–138 in M2′ and PH2′ are not shown. **e**, Comparison of the structures of halothane, isoflurane and sevoflurane. **f**, Analysis of isoflurane inhibition of WT THIK-1, THIK-1-Y273A and THIK-1-T237A. All values are shown as mean ± s.e.m. (*n* ≥ 6 for each). **g**, Summary of volatile anesthetic inhibition with either 3.0 mM halothane, 4.9 mM isoflurane and 0.24 mM sevoflurane for WT THIK-1 and mutant channels as indicated. **h**, Representative recording under conditions as in **a**, showing dose-dependent halothane inhibition for THIK-1 channels activated with 5.0 µM oleoyl-CoA. **i**, Analysis of halothane inhibition in the absence and presence of 5.0 µM oleoyl-CoA from recordings as in **a** and **h**. **j**, Fold activation of WT THIK-1 with 5.0 µM oleoyl-CoA in the absence and presence of 15.2 mM halothane. Data are shown as mean ± s.e.m., with the number (*n*) of individual recordings indicated above the bars or in the legend. Unstimul., unstimulated; pre-inh., pre-inhibition. Source data

THIK-1 derives its original name from its sensitivity to the volatile anesthetic halothane. However, the binding site for halothane and its mechanism of inhibition remain unknown. To examine whether halothane inhibition also operates via the Y gate we first tested for any direct competition with TPenA and found that halothane inhibition was reduced in the presence of TPenA (Fig. 3a,b) indicating that it may also bind within the inner cavity.

**Extended Data Fig. 3:**
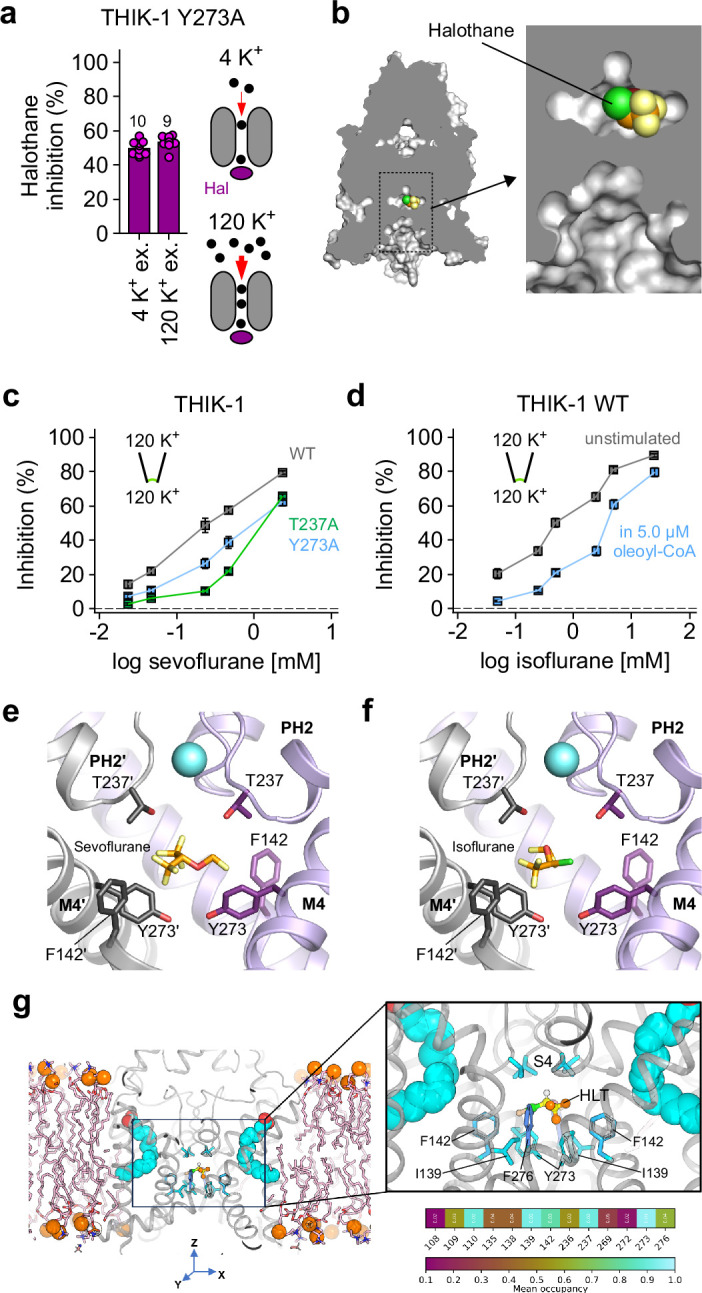
Volatile anaesthetic inhibition in THIK-1 channels. **a**, Analysis of halothane inhibition of THIK-1 channel currents recorded at −80 mV from excised patches at pH 7.4 with low (4 mM) and high (120 mM) extracellular K^+^ indicating lack of effect of external [K^+^] which would otherwise displace a pore blocker. **b**, Surface representation and expanded cutaway of THIK-1 showing halothane docked off centre within the inner cavity. **c**, Analysis of sevoflurane inhibition for WT (gray), THIK-1 T237A (green) and Y273A mutant channels (blue), respectively. **d**, Analysis of halothane inhibition in the absence and presence of 5 µM oleoyl-CoA. **e,f**, THIK-1 with docked sevoflurane (e) or isoflurane (f) in the inner vestibule. For clarity, residues 121–138 in M2’ and PH2’ are not shown. K^+^ in the S4 site is shown as a cyan sphere. **g**, Confirmation of the predicted halothane binding pocket using MD-based docking. The binding site beneath the filter is indicated by the box and expanded on the right. Residues that contact halothane (HLT) are shown as cyan sticks and linoleic acid within the modulator site shown as VdW spheres. S4 represents T110 and T237 that form the S4 K^+^ binding site. Halothane is dynamic within this binding pocket and forms contacts with residues that include those identified by mutagenesis that affect halothane activation. The mean contact occupancy with halothane is indicated in the colour scale. All values shown in this figure are given as mean ± s.e.m with number (*n*) of individual recordings indicated above the bars. Source data

In other K2P channels, TPenA interacts with the conserved threonines of the TxGYG motif at the entrance to the filter (T110 in SF1 and T237 in SF2). Substitution of either threonine reduced TPenA inhibition (Extended Data Fig. 2b), but only the T237A mutation in SF2 reduced halothane inhibition (Fig. 3c), thus suggesting an off-center, lateral binding site in this asymmetric cavity. This also implies that halothane might not directly block the THIK-1 pore. Consistent with this, changes in extracellular K^+^ concentration had no effect on the efficacy of halothane inhibition of a more open, Y gate gain-of-function mutant THIK-1 at −120 mV, indicating no knock-off effect occurs and that open channel block by halothane is unlikely (Extended Data Fig. 3a).

Using the cryo-EM structure of THIK-1, we performed initial docking studies that identified a potential halothane binding site on one side in the inner cavity between F142 on M2, Y273 on M4 and T237 in SF2 (Fig. 3d and Extended Data Fig. 3b). Substitutions in the predicted binding site affected inhibition by halothane, as well the structurally related isoflurane and sevoflurane (Fig. 3e–g and Extended Data Fig. 3c). Similar binding sites were also identified for both isoflurane and sevoflurane, suggesting a conserved binding site in the inner cavity for these volatile anesthetics (Extended Data Fig. 3e,f). To further validate this potential halothane-binding site, we used a molecular-dynamics-based approach, which identified similar contacts with halothane (Extended Data Fig. 3g).

We next found that opening of the Y gate by lipid activation reduced both halothane and isoflurane inhibition (Fig. 3h,i and Extended Data Fig. 3d), and that preinhibition of WT THIK-1 channels with halothane reduced the extent of activation by 5 µM oleoyl-CoA (14 ± 1-fold, *n* = 64 for WT versus 8 ± 2-fold, *n* = 8, in the presence of halothane) (Fig. 3i). Overall, these results indicate that halothane binding in the inner cavity dynamically regulates opening and closing of the Y gate. However, the interactions with residues in both the SF and Y gate suggest that additional effects of halothane on any SF gate cannot be excluded.

### Extracellular ion exit pathway

The extracellular cap domain above the filter results in a bifurcated exit route for K^+^. Substitutions in these pathways in other K2P channels have been shown to influence channel properties, in particular their sensitivity to blockers that interact with the keystone inhibitor site (KIS) immediately above the filter^22^, and obstruction of the pathway by nanobodies has also been shown to inhibit channel activity^23^.

**Fig. 4:**
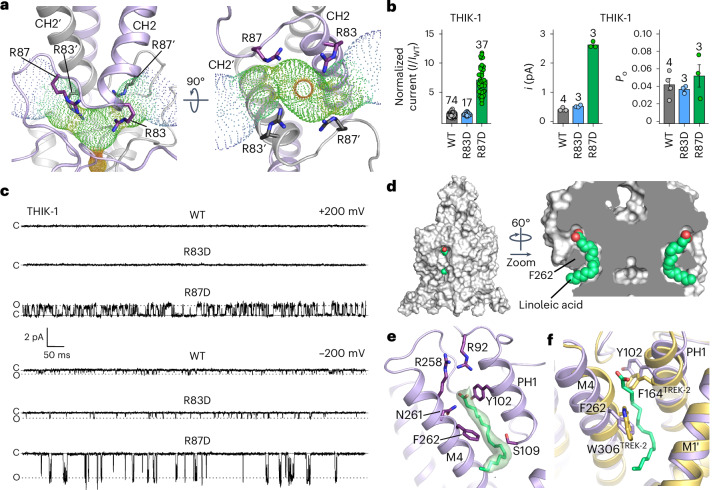
Regulation of THIK-1 activity by charged residues and lipids. **a**, Bifurcated extracellular ion exit pathway for K^+^, showing the orientation of positively charged residues in that region (R83 and R87). **b**, Left: relative whole-cell current amplitudes of WT and mutant THIK-1 channels (R83D and R87D). Right: analysis of the single-channel *i* and *P*_o_ from *n* ≥ 3 recordings, as shown in **c** for WT and mutant THIK-1 channels in cell-attached patches at −200 mV. **c**, Representative single-channel recordings of WT THIK-1 compared with channels for the R83D and R87D mutants. Recordings shown at ±200 mV in the cell-attached configuration. The closed and open channel levels are shown. **d**, Surface representation of THIK-1 and cutaway showing two linoleic acid molecules (green) in the curved lipid binding pocket. **e**, The density assigned to linoleic acid within this binding pocket is shown in green, with residues that have been altered for functional studies highlighted as sticks. **f**, Structural overlay of THIK-1 (purple) with TREK-2 (yellow), showing the position of F262 and Y102 in THIK-1, corresponding to W306 and F164 in TREK-2, which rotate during channel activation. Values are given as mean ± s.e.m., with the number (*n*) of individual recordings indicated above the bars. Source data

**Extended Data Fig. 4:**
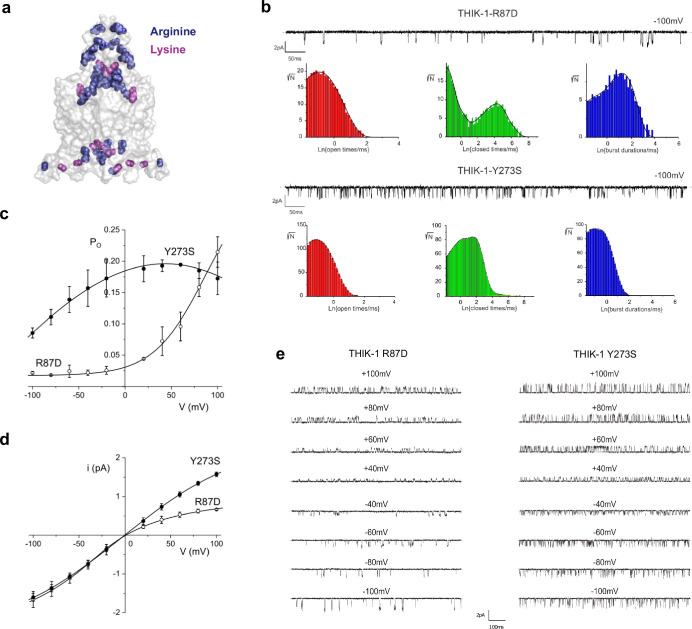
Role of positive charges in the cap domain. **a**, Transparent surface representation of THIK-1 showing the cluster of arginine (blue) and lysine (purple) residues in the cap domain and at the intracellular pore site. **b**, Comparison of single-channel kinetics of THIK-1-R87D (top) and THIK-1 Y273S (bottom) recorded in cell-attached configuration at −100 mV. Traces represent 1 s recordings with horizontal dotted lines indicating the zero current level. Below the traces are dwell time histograms of openings (red bars), closings (green bars) and bursts of openings (blue bars). The lines are probability density function fits to the data: t_1_ = 0.31 ms, A_1_ = 611; t_2_ = 0.67 ms, A_2_ = 529; (openings); t_1_ = 0.14 ms A_1_ = 902; t_2_ = 0.58 ms, A_2_ = 136; t_3_ = 0.79 ms, A_3_ = 11; t_4_ = 14 ms, A_4_ = 13; t_5_ = 66 ms, A_5_ = 184; t_6_ = 185 ms; A_6_ = 42 (closings) and t_1_ = 0.23 ms, A_1_ = 36; t_2_ = 3.5 ms, A_2_ = 204 (bursts) for THIK-1 R87D, and t=0.33 ms, A=38900 (openings) and t_1_ = 0.17 ms A_1_ = 1914; t_2_ = 0.78 ms, A_2_ = 9500; t_3_ = 5.3 ms, A_3_ = 17613; t_4_ = 11 ms, A_4_ = 1695; t_5_ = 58 ms, A_5_ = 37; t_6_ = 300 ms; A_6_ = 3 (closings) and t_1_ = 0.12 ms, A_1_ = 7900; t_2_ = 0.59 ms, A_2_ = 22976 (bursts) for THIK-1-Y273S. **c**, Comparison of voltage-dependence of single-channel open probability (P_O_) and **d**, single-channel current amplitude (i) of THIK-1 R87D and THIK-1 Y273S mutant channels (All values are given as mean ± s.e.m, *n* ≥ 3). Note, lines through the data are drawn by hand. **e**, Cell-attached recordings of 1 s duration at voltages between −100 mV and +100 mV of single THIK-1 R87D and THIK-1 Y273S, as indicated. The dotted lines represent zero current levels. Source data

In most K2Ps, the presence or absence of negative charges at the KIS affects channel conductance and pharmacology^24,25^. By marked contrast, the structure of THIK-1 revealed an unusually large number of positive charges, mostly arginine residues, distributed throughout the cap domain, several of which are the KIS and sit directly above the filter (Fig. 1e and Extended Data Fig. 4a). In particular, the R83 and R87 side chains from each subunit position four positive charges directly above the filter and the S0 K^+^-binding site, likely influencing K^+^ permeation through the filter itself and also the extracellular exit pathways that they line (Fig. 4a).

Notably, a charge reversal at the first of these positions (THIK-1-R83D) produced channels with whole-cell currents ~1.5-fold larger than that of WT THIK-1 (Fig. 4b), but still with no resolvable single-channel openings at positive potentials, and only a modest effect at inward potentials (Fig. 4c). However, a charge reversal at the second position (THIK-1-R87D) produced whole-cell currents ~7-fold larger than that of WT THIK-1, and single-channel recordings revealed a markedly increased single-channel conductance at both positive and negative voltages (Fig. 4b,c). THIK-1-R87D also exhibited kinetics that were distinct from channels with substitutions in the Y gate (THIK-1-Y273S), with openings clustered into longer bursts and clearly separated intra- and inter-burst closed states (Extended Data Fig. 4b).

Also, compared with THIK-1-Y273S, the *P*_o_ of THIK-1-R87D was steeply voltage-dependent at positive membrane potentials (Extended Data Fig. 4c,e), suggesting a negative charge at this position not only stimulates K^+^ efflux through the filter, but also promotes channel opening (that is, increases *P*_o_) by stabilizing the open conformation of the SF. Furthermore, although the *P*_o_ of the THIK-1-R87D and THIK-1-Y273S mutants approach similar values at +100 mV, R87D single channels exhibit inward rectification, unlike Y273S channels (Extended Data Fig. 4d,e). This suggests that a negative charge at position 87 can hinder the exit of K^+^ through extracellular ion exit pathways, reducing single-channel conductance at positive membrane potentials. Intriguingly, human microglia have a relatively positive resting membrane potential (−30 to −40 mV) that can be depolarized even further by selective THIK-1 inhibitors^5^. It will be interesting to determine how these positive charges in THIK-1 contribute to the unusual electrical properties of these cells.

**Extended Data Fig. 5:**
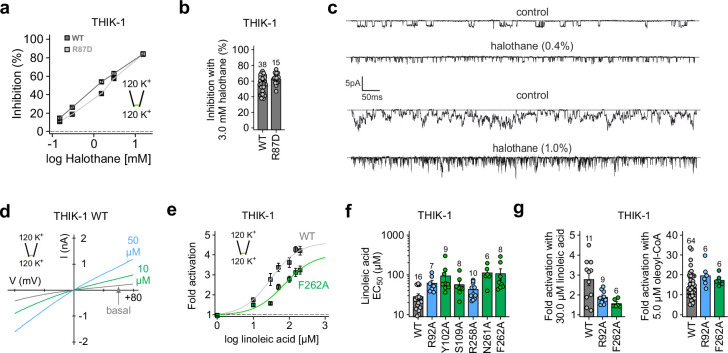
Mechanisms of halothane inhibition and lipid activation of THIK-1. **a,b**, Analysis of halothane inhibition for WT and THIK-1 R87D mutant channels, respectively showing that the R87D channel retains similar sensitivity to WT THIK-1. **c**, Excised patch recordings of 1 s duration at −100 mV of single R87D THIK-1 channels in the absence (control) and presence of 0.4 % (upper panel) or 1% halothane (lower panel). The dotted lines represent zero current levels. Halothane induces destabilisation (shortening) of both open and closed states. Thus, because destabilisation of channel closings increases *P*_o_, then destabilisation of channel openings must drive the inhibitory effect of halothane. Consistent with this, higher concentrations of halothane (1% lower panel) induced a further, more dramatic reduction in the duration of openings, resulting in ‘flickery’ single-channel kinetics and an even greater decrease in channel *P*_o_ (30% and 75% decrease in activity in the presence of 0.4% and 1% halothane, respectively. **d**, Representative recording from an inside out patch containing WT THIK-1 with symmetrical K^+^ concentrations (120 mM [K^+^]) at pH 7.4. Channel currents were activated with indicated concentrations of linoleic acid. **e**, Analysis of fold activations from recordings as in panel f for WT R92A and F262A mutants showing reduced effect on linoleic acid activation but no effect on oleoyl-CoA activation. All values shown in this figure are given as mean ± s.e.m with number (*n*) of individual recordings indicated above the bars or in the legend. **f**, EC_50_ values from dose-response curves as in panel e for WT and mutant THIK-1. **g**, Fold activation of WT and R92A and F262A mutant THIK-1 channels, respectively with either 30 µM linoleic acid or 5 µM oleoyl-CoA. Source data

The well-resolved single-channel behaviour of the R87D mutant also presents an opportunity to examine the effects of halothane inhibition in more detail, because its effects on WT THIK-1 single channels would be more challenging to assess. Excised patch experiments show comparable inhibition for WT and R87D channels (Extended Data Fig. 5a,b). Notably, we found that halothane induced destabilization (shortening) of both open and closed states, with the former seemingly driving this inhibitory effect (Extended Data Fig. 5c).

### A lipid bound within the K2P modulator pocket

A cryptic small-molecule-binding site has previously been identified in structures of TREK channels in complex with certain activators (ML335 and ML402)^26^. This pocket is located at the PH1–M4 interface, modulation of which dynamically regulates K2P channel activity through direct effects on the filter-gate^27^. It was therefore intriguing to observe density in this pocket consistent with a fatty acid (Figs. 1f and 4d–f and Extended Data Fig. 1e). Because this binding pocket is also highly curved, the bound lipid is likely to be a polyunsaturated fatty acid (PUFA), and we found that the omega-3 essential fatty acid, linoleic acid (18:2), fits well into this density (Fig. 4d–f). Two arginine side chains (R92 and R258) are in close proximity to the headgroup of this lipid, and F262 on M4 contacts the middle of the lipid (Fig. 4e). This is particularly noteworthy because in many other K2P channels, F262 is a conserved tryptophan that is predicted to rotate inward into this pocket during channel activation^23,28^ (Fig. 4f), and any lipid bound within this pocket therefore has the potential to modulate THIK-1 activity and influence microglial function.

Owing to its curved nature, the lipid-binding site shown in Fig. 4d can accommodate only a restricted range of PUFAs, with linoleic acid being the prime candidate; however, the precise identity of the lipid in this site remains to be determined unambiguously. Also, the fact that this lipid copurifies with THIK-1 indicates that it is tightly bound and could represent an important structural or functional cofactor, rather than a direct modulator itself. Nevertheless, it was intriguing to observe that linoleic acid directly activates THIK-1 when applied intracellularly to excised patches, with a half-maximal effective concentration of 29 ± 5 µM (*n* = 16), and that substitutions in this pocket reduce both the extent and apparent affinity of this activation without affecting activation by either oleoyl-CoA or PIP_2_ (Extended Data Fig. 5d–g). The functional role of this PUFA-binding site clearly warrants further investigation because microglia can sense a variety of extracellular lipids that stimulate their inflammatory response, and changes in linoleic acid and related omega lipids have been shown to influence microglial activity^29,30^. However, it remains to be determined whether dynamic regulation by linoleic acid and/or other PUFAs is physiologically relevant, or whether tightly bound PUFAs in this site are simply required to support the tonic activity of THIK-1.

## Discussion

Overall, this study provides important structural insight into the gating and permeation of this unusual subfamily of K2P channels, and reveals a unique lower cytoplasmic Y gate that responds to physiologically relevant signals. Furthermore, by providing a structural framework for the optimization of both THIK-1 inhibitors and possible activators, these results have major implications for the modulation of microglial function and future therapeutic strategies for the treatment of neurodegenerative disorders.

## Methods

### Cloning and protein expression

Human *KCNK13*, encoding residues Gly9 to Gly297, was subcloned into a modified pFastBac vector, encoding an HRV 3C protease site, a decahistidine tag and a FLAG tag, using ligase-independent cloning. Bacmid DNA was generated using the Bac-to-Bac system, and the resulting bacmid transfected into *Spodoptera frugiperda* (Sf) 9 insect cells. The resulting virus was amplified twice and used for large-scale infections of Sf9 cells at a density of 2 × 10^6^ cells ml^–1^, using 5% vol/vol virus per L of cells, and grown for 72 h at 27 °C. Cells were collected by centrifugation at 900*g* for 10 min and stored at −80 °C before protein purification.

### Purification

Cells were resuspended in 40 ml breaking buffer (50 mM HEPES pH 7.5, 200 mM KCl, 5% vol/vol glycerol) per litre of initial cell culture volume and lysed using an EmulsiFlex- C5 high-pressure homogenizer (Avestin), before solubilization in 1% wt/vol octyl glucose neopentyl glycol (OGNG) and 0.1% wt/vol cholesteryl hemisuccinate (CHS) for 1 h. Residual debris was pelleted by centrifugation at 35,000*g* for 1 h. To collect the protein, the supernatant was incubated with 1 ml 50% vol/vol Talon resin (Clontech) and 5 mM imidazole pH 8.0 per initial L of cell culture for 1 h. The resin was collected and washed with 30 column volumes of wash buffer (50 mM HEPES pH 7.5, 200 mM KCl, 5% vol/vol glycerol, 20 mM imidazole pH 8.0, 0.18% wt/vol OGNG, 0.018% wt/vol CHS), and the final volume of resin and buffer was adjusted to two column volumes. On-column cleavage and deglycosylation of the protein were performed by adding 150 µg hexahistidine-tagged HRV 3C protease and 50 µg hexahistidine-tagged PNGaseF, before overnight incubation. The flow-through was collected, concentrated to 500 µl and subjected to size-exclusion chromatography on a Superose 6 Increase 100/300 GL column (Cytiva) in gel filtration buffer (20 mM HEPES pH 7.5, 200 mM KCl, 0.12% wt/vol OGNG, 0.012% wt/vol CHS). All purification steps were performed on ice or at 4 °C.

### Cryo-EM grid preparation and data collection

Electron microscopy was provided through the Central Oxford Structural Molecular Imaging Centre (COSMIC) facility. Grids were prepared by plasma-treating holey gold grids (GF-1.2/1.3, 300 mesh, 45 nm film) and adsorbing 3 µl THIK-1 at 4 mg ml^–1^, followed by blotting for 3–6 s at 100% humidity at 4 °C. Grids were then vitrified in liquid ethane, using a Vitrobot Mark IV (Thermo Fisher Scientific). Data were collected on a Titan Krios G3 (FEI) with a K3 direct detection camera (Gatan) and a BioQuanum imaging filter (Gatan), at 300 kV in counted super-resolution bin 2 mode and ×105,000 magnification, with a pixel size of 0.832 Å and a total dose of 42.54 e^−^ Å^–2^ over 40 fractions. A total of 21,171 videos were collected over a defocus range of −1.0 to −2.3 µm.

### Data processing

Motion correction and contrast transfer function (CTF) estimation was done on a subset of 1,060 videos, using the preprocess_stream pipeline within SIMPLE^31^. Manual particle picking was followed by autopicking, extraction and one round of two-dimensional (2D) classification in SIMPLE. Particles belonging to the accepted 2D classes were imported into cryoSPARC^32^. All further processing steps were done in cryoSPARC, unless stated otherwise. The particles were subjected to two rounds of 2D classification and one round of ab initio reconstruction with five classes, and templates were generated from one of the classes. The templates were then used for repicking of the micrograph subset. The extracted particles were subjected to similar steps to generate improved templates.

The full dataset was motion corrected in SIMPLE, using the preprocess_stream pipeline, and motion-corrected micrographs were imported into cryoSPARC where they underwent patch CTF estimation. Of the initial 21,171 micrographs, 18,838 were kept, and particles were then picked from these using the previously generated templates. A total of 9,257,778 particles were extracted, with a box size of 256 pixels, and these underwent one round of unmasked 2D classification and five rounds of 2D classifications with a 125-Å spherical mask. The selected 1,240,628 particles were subjected to ab initio volume reconstruction with five classes, with no symmetry applied, followed by heterogeneous refinement. Three of the classes (878,207 particles in total) were further cleaned by one round of 2D classification, narrowing the set down to 765,828 particles. These were used for another round of ab initio reconstruction with five classes, with *C*_2_ symmetry applied, followed by heterogeneous refinement. The particles in one class (327,614), were further refined with homogeneous and non-uniform refinement, with *C*_2_ symmetry applied. They were then exported to RELION-3, using the csparc2star.py script within UCSF pyem (https://zenodo.org/records/3576630), and underwent Bayesian polishing^33^. After a final round of 2D classification, the cleaned and polished particle set (302,189) was used for non-uniform refinement, with *C*_2_ symmetry applied. A final resolution of 3.16 Å was estimated with gold-standard Fourier shell correlations using the 0.143 criterion. The map was subsequently sharpened with a *B* factor of −140.

### Model building and refinement

An initial THIK-1 model was built manually in Coot^34^. An elongated density was observed behind the filter, and we chose to model linoleic acid, in this density, on the basis of the fit to the map. The model was refined with phenix.real_space_refine^35,36^, with non-crystallographic symmetry (NCS) constraints and restraints for the linoleic acid generated using the Grade2 server, and otherwise default settings. The refined model was further improved in ISOLDE^37^, within ChimeraX^38^, and used to generate a *C*_2_ symmetric model that was subjected to real space refinement, with NCS constraints and ligand restraints as before, but without rotamer or Ramachandran restraints. The input model was also restrained to the ISOLDE-generated A chain. This model was then subjected to a final round of adp refinement. The radii of the channel pore and the extracellular ion pathways were calculated using HOLE^39^.

### Molecular docking

Initial molecular docking into this structure of THIK-1 was conducted to evaluate the most probable binding poses for halothane, isoflurane and sevoflurane. On the basis of the result of the competition assay of halothane with TPenA, it is expected that halothane binds in the pore cavity close to the known binding sites for QA blockers. Molecular docking using Pymol was used to explore possible binding poses of halothane in the structure with the potassium density at the filter S4 site, defined as the center of the docking pocket. A receptor grid was calculated for THIK-1 with a box size of 10 × 10 × 10 Å. Docking of halothane, isoflurane and sevoflurane into the pocket was then carried out. The three docking poses with the highest scores were selected and further validated by electrophysiological analysis. To further validate this site, a molecular-dynamics-based approach was also conducted, following an identical protocol previously applied to modulator-bound TREK-1 structures simulated in the POPC membrane^40^. In brief, halothane-bound THIK-1 was simulated using GROMACS 2024 and CHARMM36m with the default CGenFF parameters for halothane, and ten independent simulations, each 1 microsecond long, were generated. Halothane atoms within 0.45 nm of any heavy protein atom were considered for the occupancy analysis. Occupancies were averaged per residue, and the mean occupancy was treated as an independent sample for each of the ten simulation replicas.

### Molecular biology

Human K_2P_13.1 THIK-1 (GenBank accession number: NM_022054) and human K_2P_12.1 THIK-2 (NM_022055.1) were used in this study. For functional expression studies, the respective K^+^ channel subtype coding sequences were subcloned into the oocyte expression vector pSGEM or the dual-purpose expression vector pFAW^41^ and verified by sequencing. All mutant channels were obtained by site-directed mutagenesis and verified by sequencing. To increase surface expression and macroscopic currents, measurements of THIK-2 channels used a mutated ER retention motif, (R11A R12A R14A R15A R16A; THIK-2*)^20^. Vector DNA was linearized with NheI or MluI, and mRNA was synthesized in vitro using the SP6 or T7 AmpliCap Max High Yield Message Maker Kit (Cellscript, USA) or HiScribe T7 ARCA mRNA Kit (New England Biolabs).

### Electrophysiological measurements

Two-electrode voltage–clamp (TEVC) studies were performed in *Xenopus* oocytes. All animal use conformed to the Guide for the Care and Use of Laboratory Animals (NIH Publication 85–23), and all experiments using *Xenopus* toads were approved by the local ethics panels. Oocytes were stored at 17 °C in ND96 recording solution composed of (in mM): 96 NaCl, 2 KCl, 1.8 CaCl_2_, 1 MgCl_2_, 5 HEPES (pH 7.5 adjusted with NaOH and HCl), supplemented with Na-pyruvate (275 mg L^–1^), theophylline (90 mg L^–1^) and gentamicin (50 mg L^–1^). Oocytes were injected with 1 ng mRNA for WT or mutant channels, and were incubated for 24–48 h at 17 °C. Two-electrode voltage clamp recordings were then performed as previously described^42,43^. For macroscopic patch–clamp measurements, oocytes were obtained as described above incubated at 17 °C in a solution containing (mM): 54 NaCl, 30 KCl, 2.4 NaHCO_3_, 0.82 MgSO_4_ ×7 H_2_O, 0.41 CaCl_2_, 0.33 Ca(NO_3_)_2_ ×4 H_2_O and 7.5 TRIS (pH 7.4 adjusted with NaOH and HCl) for 1–4 days before use. Excised patch recordings in inside-out configuration conditions were performed at room temperature. Patch pipettes were made from thick-walled borosilicate glass GB 200TF-8P (Science Products), had resistances of 0.2–0.5 MΩ (tip diameter, 10–25 µm) and filled with a pipette solution (in mM): 120 KCl, 10 HEPES and 3.6 CaCl_2_ (pH 7.4 adjusted with KOH and HCl). Intracellular bath solutions and compounds were applied to the cytoplasmic side of excised patches for the various K^+^ channels through a gravity flow multi-barrel pipette system. The intracellular solution had the following composition (in mM): 120 KCl, 10 HEPES, 2 EGTA and 1 pyrophosphate (pH adjusted with KOH and HCl). Currents were recorded with an EPC10 amplifier (HEKA Electronics) and sampled at 10 kHz or higher, with filtering set to 3 kHz (–3 dB) or higher, as appropriate for sampling rate. For measurements of GPCR activation, whole-cell currents were recorded for THIK-1 in transiently transfected HEK-293 cells. Cells were stimulated by a ramp protocol between −100 and +60 mV (holding potential, −80 mV). The pipette solution was composed of (in mM): 140 KCl, 2 MgCl_2_, 1 CaCl_2_, 2.5 EGTA and 10 HEPES (pH 7.3 adjusted with KOH and HCl). The extracellular bath solution had the following composition (in mM): 135 NaCl, 5 KCl, 2 MgCl_2_, 2 CaCl_2_, 10 glucose and 10 HEPES pH (pH 7.3 adjusted with NaOH and HCl). Oxotremorine M (Oxo-M) was added to extracellular bath solution to obtain the final concentration. Single-channel currents were recorded with an Axopatch 200B amplifier through a Digidata 1440A digitizer (Molecular Devices). Data were filtered at 2 kHz and recorded at a 200-kHz sampling rate with the program Clampex (Molecular Devices). Pipette solution and bath solution for cell-attached recordings contained (in mM): 140 KCl, 2 MgCl_2_, 1 CaCl_2_ and 10 HEPES (pH 7.4 adjusted with KOH and HCl). For inside-out recordings, the bath solution contained (in mM): 140 KCl, 2 MgCl_2_, 1 CaCl_2_ and 10 HEPES (pH 7.2 adjusted with KOH and HCl). All experiments were conducted at room temperature.

### Clinical drugs, chemical compounds and lipids

TPenA, linoleic acid, l-α-phosphatidylinositol 4,5-bisphosphate (brain PI(4,5)P_2_, PIP_2_) (Sigma-Aldrich and Merck) and oleoyl-CoA (LC-CoA 18:1) (Avanti Polar Lipids) were prepared as stocks (1–100 mM) in DMSO, stored at −80 °C and diluted to the final concentration in the intracellular recording solution. Oxo-M (Tocris Bioscience,) was prepared as 50 mM stock in H_2_O. Halothane (99%) (Sigma-Aldrich/Merck), isoflurane (100%) (Baxter) and sevoflurane (100%) (AbbVie) were added to the intracellular recording solution, which was then shaken for 3 min. After clear phase separation (~5 min), the intracellular solution was used for experiments within 15 min.

### Data acquisition, statistical analysis and reproducibility

Data analysis and statistics for macroscopic measurements were done using Fitmaster (HEKA electronics, version: v2x90), Microsoft Excel 2021 (Microsoft Corporation) and Igor Pro 9 software (WaveMetrics). Recorded currents were analyzed from membrane patches at a voltage defined in the respective figure legend or with a voltage protocol as indicated in the respective figure. The fold activation (fold change in current amplitude) of a ligand (clinical drug, compound or lipid) was calculated from the following equation:

$${\rm{Fold}}\; {\rm{activation}}\left({\rm{FA}}\right)=\frac{{{{I}}}_{{\rm{activated}}}}{{{{I}}}_{{\rm{basal}}}}$$

where *I*_activated_ represents the stable current level in the presence of a given concentration of a respective ligand, and *I*_basal_ represents the measured current before ligand application. Percentage inhibition upon blocker application for a ligand was calculated from stable currents of excised membrane patches using the following equation:

$$\% {\rm{inhibition}}=\left(1-\left(\frac{{{{I}}}_{{\rm{inhibited}}}}{{{{I}}}_{{\rm{basal}}}}\right)\right)\times 100$$

where *I*_inhibited_ refers to the stable current level recorded in the presence of a given concentration of the ligand, and *I*_basal_ to the measured current before ligand application. The macroscopic half-maximal concentration–inhibition relationship of a ligand was obtained using a Hill-fit for dose–response curves, using the following equation:

$$\% {\rm{inhibition}}\ {\rm{or}}\ \%{\rm{activation}}=\frac{{I}_{\rm{base}}+\left({I}_{\max }-\,{I}_{\rm{base}}\right)}{\left\{{1+\left[\frac{{x}_{1/2}}{x}\right]}^{{H}}\right\}}$$

where *I*_base_ and *I*_max_ are the currents in the absence and presence of a respective ligand, *x* is the concentration of the ligand, *x*_1/2_ is the ligand concentration at which the activatory or inhibitory effect is half-maximal and *H* is the Hill coefficient.

For analysis of block and release from block time constants, current traces were fitted with a mono-exponential equation:

$${{y}_{0}+A}^{\left\{\frac{-(x-{x}_{0}}{\tau }\right\}}$$

Data from individual measurements were normalized and fitted independently, to facilitate averaging. A Kolmogorow–Smirnow test was used to determine whether measurements were normally distributed. Statistical significance between two groups (respective datasets) was validated using an unpaired two-tailed Student’s *t*-test. Image processing and figure design was done using Igor Pro 9 (64 bit) (WaveMetrics), PyMOL 2.4.1 (Schrödinget) and Canvas X Draw (version 20 build 544) (ACD Systems). For analysis of single channels, recordings were idealized using 50% threshold criterion with Clampfit (Molecular Devices) at an imposed resolution of 50 µs. Amplitude and dwell-time distributions were analyzed using Origin (OriginLab Corporation). Critical time for burst analysis was determined using Magleby and Pallotta criterion^44^.

### Reporting summary

Further information on research design is available in the Nature Portfolio Reporting Summary linked to this article.

## Online content

Any methods, additional references, Nature Portfolio reporting summaries, source data, extended data, supplementary information, acknowledgements, peer review information; details of author contributions and competing interests; and statements of data and code availability are available at 10.1038/s41594-025-01497-6.

## Supplementary information

Reporting Summary

## Source data

Source Data Figs. 1–4 and Extended Data Figs. 2–5 Source data.

## Data Availability

All data in this study are included in the article, and materials are available upon request. The cryo-EM model and maps of THIK-1 have been deposited in the Protein Data Bank and the EMDB database, respectively, under accession codes 9FT7 and EMD-50741. No code was developed in this study. Source data are provided with this paper.
