# Supplementary material for: Cryo-EM structure of the human THIK-1 K2P K+ channel reveals a lower Y gate regulated by lipids and anesthetics
Source: Nat Struct Mol Biol. 2025 Feb 26;32(7):1167–74. doi: 10.1038/s41594-025-01497-6 (PMC12263422; doi:10.1038/s41594-025-01497-6)
Supplement: Supplementary file 1 — Reporting Summary [file 41594_2025_1497_MOESM1_ESM.pdf]

nature portfolio

Corresponding author(s): Stephen J Tucker

Last updated by author(s): Nov 19, 2024

Reporting Summary

Nature Portfolio wishes to improve the reproducibility of the work that we publish. This form provides structure for consistency and transparency in reporting. For further information on Nature Portfolio policies, see our Editorial Policies and the Editorial Policy Checklist.

Statistics

For all statistical analyses, confirm that the following items are present in the figure legend, table legend, main text, or Methods section.

n/a	Confirmed	
☐	☒	The exact sample size (*n*) for each experimental group/condition, given as a discrete number and unit of measurement
☐	☒	A statement on whether measurements were taken from distinct samples or whether the same sample was measured repeatedly
☐	☒	The statistical test(s) used AND whether they are one- or two-sided<br>*Only common tests should be described solely by name; describe more complex techniques in the Methods section.*
☒	☐	A description of all covariates tested
☐	☒	A description of any assumptions or corrections, such as tests of normality and adjustment for multiple comparisons
☐	☒	A full description of the statistical parameters including central tendency (e.g. means) or other basic estimates (e.g. regression coefficient) AND variation (e.g. standard deviation) or associated estimates of uncertainty (e.g. confidence intervals)
☐	☒	For null hypothesis testing, the test statistic (e.g. F, t, r) with confidence intervals, effect sizes, degrees of freedom and P value noted<br>*Give P values as exact values whenever suitable.*
☒	☐	For Bayesian analysis, information on the choice of priors and Markov chain Monte Carlo settings
☒	☐	For hierarchical and complex designs, identification of the appropriate level for tests and full reporting of outcomes
☒	☐	Estimates of effect sizes (e.g. Cohen's *d*, Pearson's *r*), indicating how they were calculated

Our web collection on statistics for biologists contains articles on many of the points above.

Software and code

Policy information about availability of computer code

Data collection	Functional and structural data were collected using a range of commonly available software as described in the methods.
Data analysis	Functional data were analysed using commonly available software (Clampfit 11, Fitmaster v2x90, Igor Pro 9, Origin 2019b and Microsoft Excel 2021) as described in the methods. Structural and biochemical data were also analysed with commonly available software as described in the methods (COOT 0.9.5; PHENIX1.19.2; CryoSPARC v4.2.1; RELION-3; SIMPLE v3.0.0)

For manuscripts utilizing custom algorithms or software that are central to the research but not yet described in published literature, software must be made available to editors and reviewers. We strongly encourage code deposition in a community repository (e.g. GitHub). See the Nature Portfolio guidelines for submitting code & software for further information.

Data

Policy information about availability of data

All manuscripts must include a data availability statement. This statement should provide the following information, where applicable:

- Accession codes, unique identifiers, or web links for publicly available datasets
- A description of any restrictions on data availability
- For clinical datasets or third party data, please ensure that the statement adheres to our policy

All data within this study are included in the article and/or the Supplementary Information, and materials are available upon request. The cryoEM model and maps

of THIK-1 have been deposited in the Protein Data Bank and the EMDB database, respectively, under accession codes 9FT7 [https://doi.org/10.2210/pdb9FT7/pdb], and EMD-50741 [https://www.ebi.ac.uk/pdbe/entry/emdb/EMD-50741].

Research involving human participants, their data, or biological material

Policy information about studies with human participants or human data. See also policy information about sex, gender (identity/presentation), and sexual orientation and race, ethnicity and racism.

Reporting on sex and gender	No human participants or human data
Reporting on race, ethnicity, or other socially relevant groupings	No human participants or human data
Population characteristics	No human participants or human data
Recruitment	No human participants or human data
Ethics oversight	No human participants or human data

Note that full information on the approval of the study protocol must also be provided in the manuscript.

Field-specific reporting

Please select the one below that is the best fit for your research. If you are not sure, read the appropriate sections before making your selection.

☒ Life sciences ☐ Behavioural & social sciences ☐ Ecological, evolutionary & environmental sciences

For a reference copy of the document with all sections, see nature.com/documents/nr-reporting-summary-flat.pdf

Life sciences study design

All studies must disclose on these points even when the disclosure is negative.

Sample size	No statistical methods were used to predetermine sample sizes. Required experimental sample size were estimated based on previous established protocols in the field and the design of the experiment. The sample sizes were adequate as the differences between experimental groups were reproducible.
Data exclusions	No exclusion criteria were pre-established. No data was excluded from the measurements shown.
Replication	The number of biological replicates (n) are illustrated in the respective figures, figure legends, graphs or tables where relevant.
Randomization	Not considered relevant for this type of experimental study due to its design.
Blinding	No animal or human populations with individual assigned to groups were used and so it is not considered relevant to do double blind tests for this type of design of experimental study.

Reporting for specific materials, systems and methods

We require information from authors about some types of materials, experimental systems and methods used in many studies. Here, indicate whether each material, system or method listed is relevant to your study. If you are not sure if a list item applies to your research, read the appropriate section before selecting a response.

Materials & experimental systems

n/a	Involved in the study	
☒	☐	Antibodies
☐	☒	Eukaryotic cell lines
☒	☐	Palaeontology and archaeology
☐	☒	Animals and other organisms
☒	☐	Clinical data
☒	☐	Dual use research of concern
☒	☐	Plants

Methods

n/a	Involved in the study	
☒	☐	ChIP-seq
☒	☐	Flow cytometry
☒	☐	MRI-based neuroimaging

Eukaryotic cell lines

Policy information about cell lines and Sex and Gender in Research

Cell line source(s)	HEK293 cells purchased from Merck (Sigma Aldrich)
Authentication	Cell lines were not authenticated by user.
Mycoplasma contamination	Tested negative
Commonly misidentified lines (See ICLAC register)	No commonly misidentified cell lines were used in this study

Animals and other research organisms

Policy information about studies involving animals; ARRIVE guidelines recommended for reporting animal research, and Sex and Gender in Research

Laboratory animals	No live animals were used directly in this study. All oocytes used were obtained in accordance with Care and Use of laboratory Animals (NIH Publication 85-23)
Wild animals	No wild animals used in this study
Reporting on sex	Only mature female Xenopus (1-3 yr old) were used for oocytes
Field-collected samples	No field collected samples
Ethics oversight	Where required, all procedures involving Xenopus were approved by veterinarians in accordance with Care and Use of laboratory Animals (NIH Publication 85-23), and relevant ethics panels (Europa und Verbraucherschutz, IX 555 - 106759/2023 33-6/23 V)

Note that full information on the approval of the study protocol must also be provided in the manuscript.

Plants

Seed stocks	No plants used
Novel plant genotypes	No plants used
Authentication	No plants used
